# Challenges in Implementing the Competency-Based Medical Education Curriculum in Pharmacology: Impact of Shortened Duration and Reduced Teaching Hours

**DOI:** 10.7759/cureus.78660

**Published:** 2025-02-06

**Authors:** Ramesh Bannaravuri, Alphienes Stanley Xavier, Rajeswari Kathiah, Anandhalakshmi S

**Affiliations:** 1 Pharmacology, All India Institute of Medical Sciences, Madurai, Madurai, IND; 2 Pathology, All India Institute of Medical Sciences, Madurai, Madurai, IND; 3 Physiology, All India Institute of Medical Sciences, Madurai, Madurai, IND

**Keywords:** cbme, curriculum implementation, faculty training, infrastructure, new elements, pharmacology, teaching hours & duration

## Abstract

Background

The Competency-Based Medical Education (CBME) curriculum was introduced by the Medical Council of India in 2019 to enhance the quality of Indian medical graduates (IMGs). Its goal is to produce IMGs who possess the knowledge, skills, attitudes, and values needed to serve as competent community physicians while remaining globally relevant. The curriculum incorporates new elements and refined assessment methods. However, the duration of the second-year MBBS course was reduced from 18 months to 12 months, and pharmacology teaching hours were cut from 300 to 230. These changes have raised concerns about the challenges of implementation and the adequacy of training. This study seeks to identify the obstacles faced by pharmacology faculty in implementing the CBME curriculum, particularly focusing on the effects of the shortened course duration, reduced teaching hours, and faculty-related challenges while proposing actionable solutions for effective implementation.

Materials and methods

This nationwide cross-sectional study, conducted between September and October 2023, surveyed 52 pharmacology faculty members from eight states and one union territory in India. Following Institutional Ethics Committee (IEC) approval number IEC-43/2022, data were collected using a structured questionnaire designed to capture faculty experiences and opinions on the CBME curriculum. Key areas of focus included course duration, teaching hours, new curriculum elements, faculty adequacy, and training status. The questionnaire, validated by internal and external experts, underwent a pilot study prior to its distribution via Google Forms (Google LLC, Menlo Park, California, USA) for purposive sampling. Responses, comprising closed-ended and Likert scale items, were analyzed using Microsoft Excel (Microsoft Corporation, Redmond, Washington, USA), with the chi-square test applied for significance. An open-ended section was included to gather additional insights for enhancing CBME implementation.

Results

Thirteen (25%) of the study participants had not undergone the Curriculum Implementation Support Program (CISP) even three years after the CBME curriculum was introduced. A majority, 37 (71.2%), felt that the reduction of the second-year MBBS duration from 18 months to 12 months and the decrease in theory hours from 100 to 80 were insufficient for effective syllabus completion. However, nearly the same percentage of faculty expressed satisfaction with the allocation of 150 practical hours, compared to the 200 hours in the pre-CBME curriculum. Notably, 45 (87%) of the faculty reported significant challenges in conducting self-directed learning, small group discussions, seminars, tutorials, and similar activities. Furthermore, all participants unanimously agreed that the inability to appoint additional assistant professors in a department, as restricted by the National Medical Commission (NMC) guidelines, to compensate for the shortage of tutors had negatively impacted the implementation of the CBME curriculum.

Conclusions

The CBME curriculum marks a significant breakthrough in the Indian medical education system. However, it is crucial to identify and assess the barriers to its effective implementation and establish a systematic feedback mechanism to ensure that the CBME curriculum, as outlined in the NMC document, is successfully put into practice.

## Introduction

The Competency-Based Medical Education (CBME) curriculum, introduced by the former Medical Council of India (now National Medical Commission (NMC)) in 2019, was designed with the goal of developing Indian medical graduates (IMGs) who possess the essential knowledge, skills, attitudes, values, and responsiveness to function effectively as community physicians while maintaining global relevance [1]. To achieve this, several new elements were incorporated into the course, including a foundation course, early clinical exposure, self-directed learning, attitude ethics and communication, alignment and integration, logbooks and reflections, electives, skills modules, a pandemic module, the learner-doctor model of clinical teaching, and a family adoption program, along with refined evaluation and assessment methods [2].

To enable faculty to effectively implement the CBME curriculum, the Medical Education Unit was restructured and strengthened. Various faculty development programs, such as the Basic Course in Biomedical Research (BCBR), Revised Basic Medical Education Training (RBMET), Curriculum Implementation Support Program (CISP), and Advanced Course in Medical Education (ACME), were introduced. These initiatives aim to equip teachers with the skills to perform multidimensional roles, including facilitator, planner, manager, performance assessor, researcher, and mentor, in addition to their traditional role as educators [3]. With these groundbreaking reforms, medical graduates from Indian institutions are expected to play critical roles as clinicians, communicators, leaders, lifelong learners, and true professionals in society [1,4,5].

However, the reduction in the duration of the second-year MBBS course from 18 months to 12 months, along with the decrease in pharmacology teaching hours from 300 to 230, presents significant challenges. This 33% reduction in course duration and approximately 25% reduction in teaching hours, compounded by the introduction of many new competencies compared to the previous curriculum, makes it increasingly difficult to implement the CBME curriculum effectively.

This study evaluates the challenges encountered in implementing the CBME curriculum in pharmacology within the constraints of reduced teaching hours and course duration. It also explores potential solutions to overcome these barriers.

Objectives

The objectives of the study were as follows: (1) to assess the adequacy of course duration and teaching hours; (2) to evaluate the effectiveness of new curriculum elements, such as self-directed learning and small group discussions; (3) to examine faculty preparedness and infrastructure challenges; and (4) to propose strategies for improving the implementation of the CBME curriculum.

## Materials and methods

Study design and location

This nationwide cross-sectional study was conducted at the All India Institute of Medical Sciences Madurai between September and October 2023, involving 52 pharmacology faculty members from eight states and one union territory in India. Informed consent was obtained from all participants, and confidentiality was maintained throughout the study. The Institutional Ethics Committee (IEC) approval for the study was granted under approval number IEC-43/2022.

Study tool

A single structured questionnaire, validated by both internal and external experts, was used to collect data. The questionnaire contained both closed-ended and open-ended questions to capture faculty experiences and suggestions. It addressed four key aspects of CBME: (i) the duration of the second-year MBBS course and the number of teaching hours in pharmacology; (ii) the new elements of the course and barriers to their implementation; (iii) the adequacy of faculty strength; and (iv) the status of faculty training in medical education [4]. An open-ended question was included at the end to allow participants to provide additional suggestions for the effective implementation of CBME. The questionnaire was self-prepared, drawing on relevant literature and input from medical education experts, and validated by two internal and two external experts in pharmacology. Reliability was assessed during the pilot phase to ensure consistency in responses. A pilot study was conducted with 10 faculty members who were not part of the main study.

Study population

The questionnaire was distributed via Google Forms (Google LLC, Menlo Park, California, USA) to pharmacology faculty across eight states and one union territory in India. The responses were then exported to Microsoft Excel (Microsoft Corporation, Redmond, Washington, USA) for analysis.

Inclusion and exclusion criteria

The inclusion criteria for the study required pharmacology faculty members actively teaching in medical colleges across India. Faculty members needed to have a minimum of three years of teaching experience and must have taught both the pre-CBME and CBME curricula. Tutors were excluded, as the study specifically focused on faculty members responsible for curriculum implementation.

The exclusion criteria eliminated faculty from institutions outside India and those without prior experience teaching under the CBME curriculum. These criteria ensured that participants had sufficient experience and relevance to the research focus.

Data analysis

The responses to the closed-ended questions, including yes/no answers and those on a 3-point Likert scale, were expressed as percentages. The chi-square test was applied to calculate the p-value. Responses to the open-ended questions were analyzed thematically. Using a qualitative content analysis approach, recurring themes were identified, with a focus on faculty perceptions and actionable feedback. This analysis complemented the quantitative data, providing a comprehensive understanding of the challenges faced in implementing the CBME curriculum.

## Results

The study revealed that 71.2% of participants found the 12-month duration for the second MBBS course inadequate for implementing the CBME curriculum (p=0.0023). Similarly, 73.1% stated that 80 theory hours were insufficient to cover the pharmacology syllabus (p=0.0009). However, 69.2% of participants felt that 150 hours allocated for practicals, small group discussions (SGDs), and self-directed learning (SDL) were adequate (p=0.0055). Overall, 78.8% believed that the CBME curriculum could not be effectively implemented with the shortened duration and reduced teaching hours in pharmacology (p=0.0001) (Table 1).

**Table 1:** Participants’ response on the duration of II MBBS and pharmacology teaching hours SDL=Self-Directed Learning; CBME=Competency Based Medical Education

Question	Response	Number (%)	Chi -Square value	p-Value
Is the duration of 12 months for Second MBBS as per CBME 2019 enough to implement the curriculum?	No	37 (71.2)	9.29	0.0023
	Yes	15 (28.8)		
Are the number of theory hours allotted (80) are adequate to cover the syllabus completely in Pharmacology as per CBME?	No	38 (73.1)	11.02	0.0009
	Yes	14 (26.9)		
Are the number of hours (150) allotted to practical/ Small Group Discussion/ Tutorials/ SDL, seminars etc. are adequate to cover the concerned syllabus in Pharmacology as per CBME?	No	16 (30.8)	7.70	0.0055
	Yes	36 (69.2)		
Do you think the CBME curriculum in its entirety is implementable effectively with shortened II MBBS duration from 18 months to 12 months and reduced number of hours from 300 to 230 in Pharmacology?	No	41 (78.8)	15.14	0.0001
	Yes	11 (21.2)		

The study highlighted significant barriers in implementing SDL, small group discussions, tutorials, and seminars, with 86.5% of participants reporting challenges (p=0.0001), as shown in table 2. The primary barriers included a lack of adequate infrastructure and faculty (86.7%), time constraints (40%), and limited student interest (31.1%). Implementation of logbooks, feedback, and reflection was rated as "somewhat effective" by 63.5% of participants, while only 9.6% found it effective (p=0.0001). Teaching space was deemed manageable by 57.7%, but 13.5% considered it grossly inadequate (p=0.0004). While 57.7% of participants supported the inclusion of National Health Programs in pharmacology, theory (48.1%) and logbook-related tasks (55.8%) were identified as the most challenging aspects of implementing CBME (p=0.0001). Alignment and integration efforts were rated "somewhat effective" by 61.5% of participants, with time constraints (51.9%) and inadequate faculty training (27%) cited as key reasons (p=0.0086). Notably, the division of the syllabus into competencies led to an equal split in opinions, with 50% agreeing and 50% disagreeing about its impact on continuity. The primary issues faced during CBME implementation were faculty shortages (67.3%) and poor interdepartmental coordination (46.2%, p=0.0002) (Table 2). 

**Table 2:** Study participants’ response on new elements of the course and barriers for implementation SDL: Self Directed Learning, AETCOM: Attitude Ethics and Communication, RBMET: Revised Basic Medical Education Training, BCW: Basic Course Workshop, rBCW: revised Basic Course Workshop, CISP: Curriculum Implementation Support Program

Question	Response	Number (%)	Chi-square value	p-Value
Are there any barriers encountered in implementation of SDL, Small Group Discussions, Tutorials, Seminars etc?	Yes	45 (86.5)	15.14	0.0001
	No	7 (13.5)		
If "yes", what are the main barriers? (n=45)				
Lack of adequate infra structure and faculty		39 (86.7)	15.14	0.0001
Lack of time		18 (40.0)		
Lack of students' interest		14 (31.1)		
Lack of faculty’s interest due to teaching overload		12 (26.7)		
How effectively were you able to implement logbook, feedback & reflection on a scale of 3?				
Not effective		14 (26.9)	15.14	0.0001
Somewhat effective		33 (63.5)		
Effective as per the guidelines		5 (9.6)		
Number of teaching areas (space and facility) available in your department/ institution for Small Group Teaching/ tutorials etc:				
Grossly inadequate		7 (13.5)	12.53	0.0004
Manageable		30 (57.7)		
Adequate		15 (28.8)		
Do you think inclusion of National Health Programs is necessary in Pharmacology as per the CBME curriculum?	No	22 (42.3)	1.23	0.2673
	Yes	30 (57.7)		
What was the most difficult part of implementing the CBME curriculum with the reduced number of hours?				
Theory		25 (48.1)	15.14	0.0001
Practical/ Tutorials/ Small Group Discussions		6 (11.5)		
AETCOM		10 (19.2)		
Logbook, Feedback & Reflections		29 (55.8)		
SDL		10 (19.2)		
How effectively the Alignment and Integration was implemented in your department?				
Not effective		11 (21.2)	15.14	0.0001
Somewhat effective		32 (61.5)		
Effective		9 (17.3)		
If the answer was "not effective"/ "somewhat effective", what was the reason identified?				
Time constraints		27 (51.9)	6.9	0.0086
Inadequate training for faculty in Medical Education		14 (27.0)		
Inadequate number of faculty		27 (51.9)		
Lack of interest by faculty due to teaching overload		16 (30.8)		
Do you think breaking of the syllabus into competencies has led to missing of the continuity and entirety?	No	26 (50.0)	0	1
	Yes	26 (50.0)		
What were the major issues faced during the implementation of CBME?				
Curriculum related		14 (27.0)	13.83	0.0002
Shortage of faculty		35 (67.3)		
Poor interdepartmental coordination		24 (46.2)		
Non completion of RBMET/rBCW/BCW/CISP training for faculty		18 (34.7)		

The findings reveal that 82.7% of participants reported that the current faculty pattern, as per the Minimum Standard Requirements set by the NMC, is inadequate for implementing the CBME curriculum (p = 0.0001). Additionally, all participants (100%) agreed that the NMC guideline prohibiting the appointment of excess assistant professors to compensate for a shortage of tutors has negatively impacted the implementation of the CBME curriculum (p = 0.0001) (Table 3).

**Table 3:** Study participants’ response on adequacy of faculty strength CBME, Competency-Based Medical Education; NMC, National Medical Commission

Question	Response	Number (%)	Chi-square value	p-value
The present faculty pattern as per the Minimum Standard Requirements by NMC is adequate to implement the CBME curriculum.	No	43 (82.7)	15.14	0.0001
	Yes	9 (17.3)		
As per the NMC guidelines, excess assistant professors in the concerned department are not allowed to compensate for the deficiency of tutors. This has adversely affected the implementation of the CBME curriculum.	Agree	52 (100.0)	15.14	0.0001
	Disagree	0 (0.0)		

The results from Table 4 show that 88.5% of participants had completed the Basic Course Workshop (BCW/RBMET/rBCW), while 11.5% had not. Similarly, 75% of participants had undergone the CISP, but 25% had yet to complete this essential training.

**Table 4:** Study participants’ status of completion of faculty development programs BCW, Basic Course Workshop; CISP, Curriculum Implementation Support Program; rBCW, revised Basic Course Workshop; RBME, Revised Basic Medical Education Training

Characteristic	Completed the training course	Number (%)
Basic Course Workshop (RBMET/BCW/rBCW)	Yes	46 (88.5)
	No	6 (11.5)
CISP	Yes	39 (75.0)
	No	13 (25.0)
Total		52 (100)

## Discussion

This cross-sectional study explores faculty opinions and experiences regarding the new CBME curriculum, focusing on various aspects such as content, structure, duration of the II MBBS course, pharmacology teaching hours, faculty preparedness, infrastructure, and overall faculty strength.

Additionally, 87% of faculty members reported difficulties in conducting self-directed learning sessions and other interactive educational formats due to inadequate infrastructure and faculty shortages. These findings reveal a significant gap between the curriculum’s requirements and the current educational environment, which may hinder effective implementation. The unanimous feedback from participants regarding the NMC’s policy restricting the use of excess assistant professors to address tutor shortages further underscores a major barrier to the successful implementation of the CBME curriculum [6-8].

Enhancing the quality of the curriculum and building faculty capacity is crucial. Faculty training programs, such as the CISP, are vital for the effective implementation of CBME. However, the pace at which faculty training programs like CISP are being carried out is concerning. Our findings show that 25% of faculty members have not completed CISP training three years after the implementation of CBME, highlighting the urgent need for the NMC to expedite these programs to meet the intended curriculum goals.

Steinert et al. (2016) in their systematic review emphasized the positive outcomes of faculty training, including enhanced personal confidence, improved skills, and increased educational leadership. Their findings suggest that while individual teaching capabilities are significantly enhanced, there is an enormous opportunity to extend these impacts to broader organizational changes [9]. Furthermore, the need for faculty training to integrate new curricula reflects similar challenges and reinforces the importance of developmental programs in promoting effective curriculum implementation. Sahadevan et al. (2021) examined specific challenges in adapting CBME in undergraduate psychiatric training in India, noting obstacles and opportunities that align with our findings [10].

This study highlights several significant challenges in implementing CBME, such as reduced teaching hours, faculty shortages, and inadequate infrastructure. Faculty training emerged as a critical factor, with 25% of participants not having completed the CISP three years after CBME’s introduction. These findings are consistent with previous studies, including Gopalakrishnan et al. (2022), which underscored the need for improved faculty development and resource allocation. Addressing these gaps is crucial for achieving the goals of the CBME curriculum [11].

Limitations

Our study has several limitations. For instance, the sample may not fully capture the diversity of faculty experiences across different types of institutions and geographic locations in India. This constraint could limit the generalizability of our findings to all educational settings. Additionally, the cross-sectional design provides insights only at a specific point in time, limiting our ability to observe trends or establish causal relationships. Furthermore, reliance on self-reported data, while valuable for capturing personal experiences and perceptions, may introduce biases related to social desirability or recall. To address these limitations, future research should aim for a more diverse and extensive sample, incorporate objective measurements, and adopt longitudinal designs. Such an approach would offer a deeper understanding of the ongoing changes and long-term impacts of CBME, improving its adaptation and effectiveness across various educational environments.

## Conclusions

The CBME curriculum marks a significant advancement in the Indian medical education system. However, its effective implementation is hindered by several challenges, including reduced course duration and teaching hours, existing faculty patterns, faculty shortages, inadequate preparedness, limited infrastructure, and the NMC’s restrictions on hiring excess assistant professors to compensate for tutor shortages. To overcome these challenges, the NMC should enhance faculty training, adopt flexible hiring policies, and allocate the necessary resources to institutions to enable them to meet the objectives of CBME effectively. Addressing these issues is essential for realizing the vision outlined in the NMC document, which aims to shape IMGs into clinicians, communicators, leaders, lifelong learners, and true professionals on the global stage.
